# Biodegradation of Photocatalytic Degradation Products of Sulfonamides: Kinetics and Identification of Intermediates

**DOI:** 10.3390/ijms25126688

**Published:** 2024-06-18

**Authors:** Daria Madej-Knysak, Ewa Adamek, Wojciech Baran

**Affiliations:** Department of General and Analytical Chemistry, Medical University of Silesia, Jagiellońska 4, 41-200 Sosnowiec, Poland; dmadej@sum.edu.pl (D.M.-K.); eadamek@sum.edu.pl (E.A.)

**Keywords:** sulfonamides, photocatalysis, kinetics, degradation products, biodegradability

## Abstract

Sulfonamides can be effectively removed from wastewater through a photocatalytic process. However, the mineralization achieved by this method is a long-term and expensive process. The effect of shortening the photocatalytic process is the partial degradation and formation of intermediates. The purpose of this study was to evaluate the sensitivity and transformation of photocatalytic reaction intermediates in aerobic biological processes. Sulfadiazine and sulfamethoxazole solutions were used in the study, which were irradiated in the presence of a TiO_2_-P25 catalyst. The resulting solutions were then aerated after the addition of river water or activated sludge suspension from a commercial wastewater treatment plant. The reaction kinetics were determined and fifteen products of photocatalytic degradation of sulfonamides were identified. Most of these products were further transformed in the presence of activated sludge suspension or in water taken from the river. They may have been decomposed into other organic and inorganic compounds. The formation of biologically inactive acyl derivatives was observed in the biological process. However, compounds that are more toxic to aquatic organisms than the initial drugs can also be formed. After 28 days, the sulfamethoxazole concentration in the presence of activated sludge was reduced by 66 ± 7%. Sulfadiazine was practically non-biodegradable under the conditions used. The presented results confirm the advisability of using photocatalysis as a process preceding biodegradation.

## 1. Introduction

Sulfonamides (SNs) are synthetic antibiotics with a bacteriostatic and herbicidal effect. Nowadays, their application in medicine has been significantly reduced, however, they are still widely used in veterinary medicine and animal husbandry. In Europe in 2022, 419 tons of SNs were consumed in veterinary medicine alone [1]. Therefore, the major source of environmental SN pollution is wastewater from livestock farms. Wang et al. [2] confirmed the presence of four SNs in swine manure in Zhejiang (China) at concentrations ranging from 9.35 to 46.37 mg/kg on a dry matter basis. An et al. [3] found that 18 mg/kg of sulfamethoxazole (SMX) on a dry matter basis was contained in manure. According to Thiebault [4], the maximum reported concentration of SMX in livestock wastewater was 54.83 mg/L in Tisumu, Kenya. High concentrations of SMX up to 1.340 mg/L were detected in wastewater from pharmaceutical production in Taiwan [5]. However, SN concentrations in wastewater typically do not exceed a few micrograms of the substance per liter, while in surface water, they are present in nanograms per liter [6,7,8,9,10,11,12]. A comprehensive summary of the data on drug occurrence in various defined aquatic ecosystems was presented in a review paper by Petel et al. [13].

SNs, which are characterized by their high polarity, mainly accumulate in the hydrosphere and can remain in the environment for a very long time [9,14]. SNs found in the biosphere can significantly affect the diversity and abundance of environmental microorganisms and induce changes in their enzymatic activity. This contributes to the imbalance of ecosystems [6,7,8,9,10,15]. More importantly, subinhibitory concentrations of SNs exert selection pressure and can cause environmental bacteria to acquire resistance traits to these drugs. In addition, the high density of microorganisms in wastewater makes the processes of acquiring resistance very intense. Resistance genes present in environmental bacteria can be transferred to human pathogens, posing a significant risk to human health [6,10,16,17]. According to the Comprehensive Antibiotic Resistance Database published by the National Center for Biotechnology Information, the prevalence of resistance to SNs is very high among pathogenic bacteria. Up to 60% of the pathogen groups tested carry a minimum of one resistance gene to this group of drugs [10].

The acquisition of drug resistance by environmental microorganisms is becoming a very substantial problem, especially considering that wastewater treatment systems currently remove SNs incompletely. Only about 20% of the used drugs in this group is estimated to be effectively eliminated [18]. These data are confirmed by the frequency of SN detection in tested environmental samples [6,7,9]. Hazard quotient (HQ) values >> 1 for SMX to algae in the effluent from WTTP were reported in Spain and in China [19,20]. This indicates the strong ecotoxic effects of inefficiently treated effluent to these organisms. Therefore, the prospect for effective methods of eliminating SNs from wastewater is very important. To degrade these drugs, studies with the use of chemical methods (reactions with H_2_O_2_, O_3_, peroxosulphates, Cl_2_, ClO_2_ and Fe(VI) compounds, and Fenton-type processes), physical methods (UV- and gamma-initiated photolysis), physicochemical methods (photocatalysis and anodic oxidation), and combined methods have been conducted [6,13,21,22,23,24,25,26]. The photocatalytic process is one of the methods proposed to degrade SNs in the aqueous environment [14,22,26,27,28,29,30,31,32,33]. Additionally, the use of this process for the pretreatment of wastewater containing antibiotics has the influence of reducing the formation of potentially drug-resistant microorganisms or genes. However, after pretreatment, wastewater may still contain some amounts of SNs and their biologically active transformation products [14,22,34,35].

The aim of the study was to apply a photocatalytic process carried out in an aqueous solution in the presence of TiO_2_-P25 to the partial degradation of selected SNs to identify the products of this process and to determine what transformations these products undergo after contact with river water or activated sludge (AS) from a wastewater treatment plant (WWTP). We suppose that the results of the presented research will allow us to evaluate the effectiveness of biological methods at further stages of wastewater treatment, as well as to make a preliminary assessment of the potential danger associated with the introduction of insufficiently treated wastewater into the hydrosphere.

## 2. Results and Discussion

### 2.1. Kinetics of SN Photocatalytic Degradation

**Figure 1:**
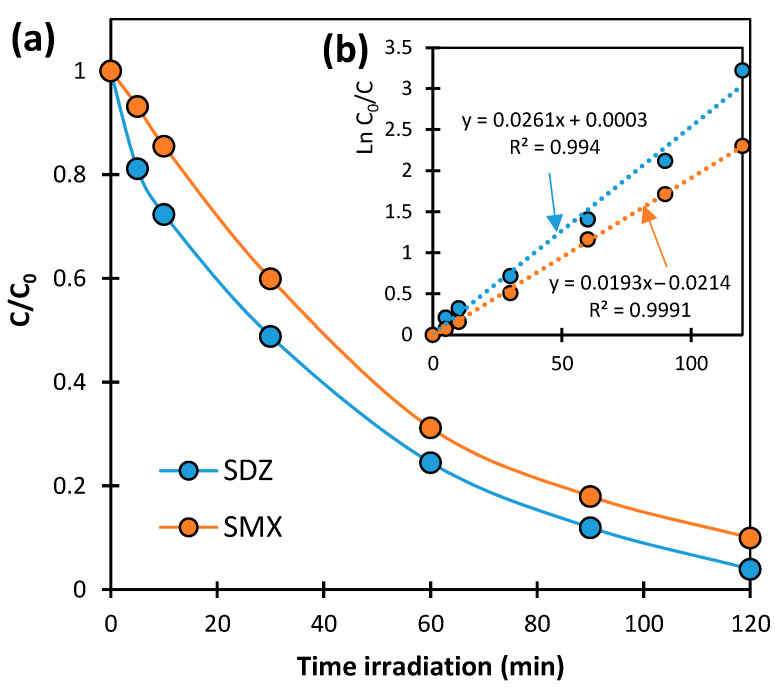
Dynamics of photocatalytic degradation of SDZ and SMX in solutions irradiated in the presence of TiO_2_-P25 (**a**) and the relationship *ln(C*_0_*/C) = f(t)* as a linear function (**b**).

The dynamics of concentration changes in SNs in solutions irradiated in the presence of TiO_2_-P25 at pH 7.0 ± 0.2 are presented in Figure 1a. The concentrations of both SNs decreased during irradiation. SDZ degrades at a slightly faster rate than SMX. After 120 min of irradiation, the degradation degrees of SDZ and SMX were 96.0 ± 2.2% and 90.0 ± 3.0%, respectively. The plots of function ln(C_0_/C) = f(t) were linear with determination coefficients R^2^ > 0.99 (Figure 1b). This confirms that the degradation of tested SNs involving TiO_2_-P25 corresponds to pseudo-first-order kinetics. The values of the photocatalytic degradation rate constants of SDZ and SMX are 0.0261 ± 0.0008 min^−1^ and 0.0193 ± 0.0002 min^−1^, respectively.

The classification of the photocatalytic degradation of SNs as a pseudo-first-order reaction has also been confirmed in other studies [14,22,31,33]. It should be mentioned that the efficiency of the discussed process cannot be directly compared with the results obtained in other studies. The photocatalytic reaction pathway depends on many factors, such as the type and concentration of catalyst, the reactant concentrations, dose and wavelength of irradiation, reactor construction, pH, etc. In most of the described studies, the required irradiation time to achieve a 90% degradation of SNs ranged from 15 to 480 min [26].

Based on the degradation rate analysis, the exposure time required to obtain solutions with the assumed concentrations of SNs undergoing subsequent biodegradation was determined. SDZ and SMX concentrations of about 0.06 mmol/L were obtained after irradiating the solutions for 60 and 75 min, respectively.

### 2.2. Kinetics of SN Aerobic Biodegradation

The solutions, after the photocatalytic process, were mixed with river water or AS in the ratio of 1:1. SDZ and SMX concentrations in the obtained solutions were approximately 0.03 mmol/L. Such concentrations of the tested SNs should not exert direct toxic effects on microorganisms present in river water or AS [14,36,37]. 

**Figure 2:**
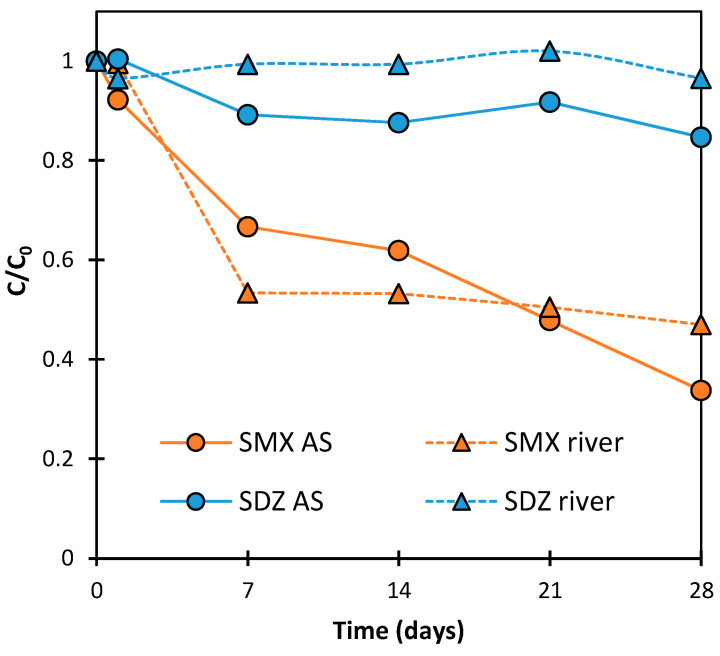
Changes in SDZ and SMX concentrations in solutions with river water or AS during aeration.

**Figure 3:**
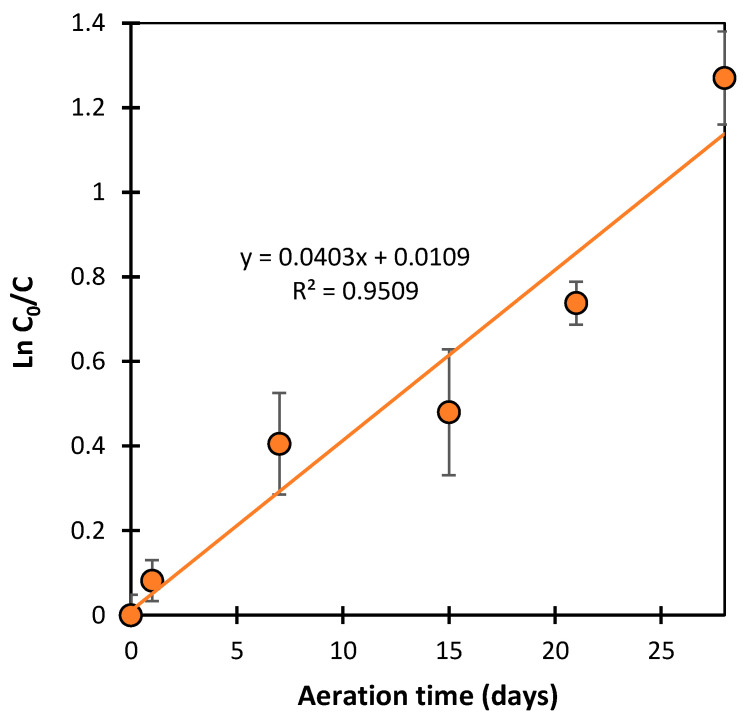
The plot of the function ln(*C*_0_/*C*) = *f*(*t*) for changes in SMX concentration in aerated solutions with AS.

Figure 2 presents the changes in SDZ and SMX concentrations during the aeration of solutions for 28 days. It was found that, after 28 days, the SDZ concentration in river water and AS suspension decreased by 4 ± 2% and 15 ± 7%, respectively. The small reduction in SDZ concentration can only be explained by this antibiotic sorption at the catalyst surface. Thus, it can be concluded that SDZ was practically not degraded under the experimental conditions. However, SMX concentration in the presence of AS was reduced by 66 ± 7%. This was most likely due to its biodegradation. The dynamics of this process corresponded to a first-order kinetics and did not show an adaptation stage (Figure 3). A decrease in SMX concentration was also observed in the solution with river water. After 28 days, the SMX concentration was reduced by 53 ± 9%. However, the dynamics of these changes in antibiotic concentration were not as expected. The initial significant loss of SMX may have been due to the biodegradation process or due to abiotic binding by an unknown component of the mixture (e.g., clay particles). The concentration of SMX practically remained constant in samples analyzed between days 7 and 28 of aeration (Figure 2). The lack of changes in antibiotic concentration could have been caused by sorbent saturation or inactivation of the microorganisms responsible for degradation after prolonged contact with SMX. 

The assessments of the applicability of biodegradation for the removal of SNs are very divergent. According to Wang et al. [38], Acinetobacter sp. is able to completely remove SMX and SDZ from wastewater in an aerobic process in 5 and 10 h, respectively. On the other hand, anaerobic microorganisms required as much as 150 days to remove more than 80% of SMX from the solution with a concentration of 15.0 mg/L [39].

### 2.3. Identification and Transformations of Products of SN Photocatalytic Degradation

The compounds present in mixtures obtained immediately after combining the photocatalytic degradation of SDZ and SMX solutions with river water or AS, as well as the products formed after 28 days of aeration of these mixtures, were identified. Therefore, it was possible to assess the stability of the products of the photocatalytic process if they were introduced into the biological WWTP or directly into the river.

**Figure 4:**
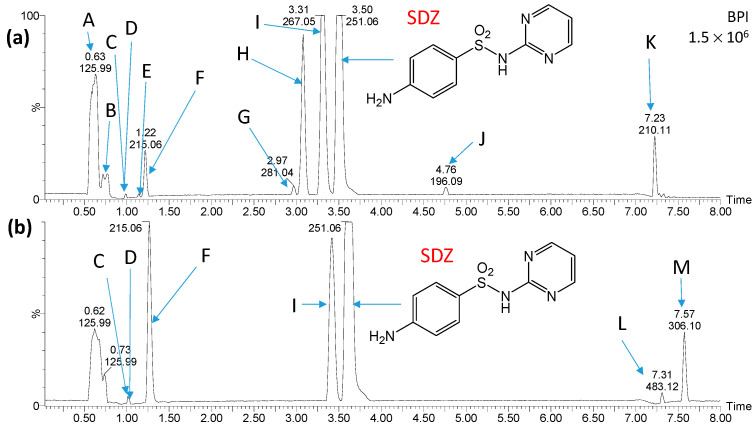
Chromatograms of the solution containing products of SDZ photocatalytic degradation shortly after mixing with river water (**a**) and after aeration of the solution for 28 days (**b**). Peaks marked with letters A, B, K, L, and M were recorded in river water samples. Structures of compounds marked with the letters C, D, E, F, G, H, I and J are shown in Figure 6.

**Figure 5:**
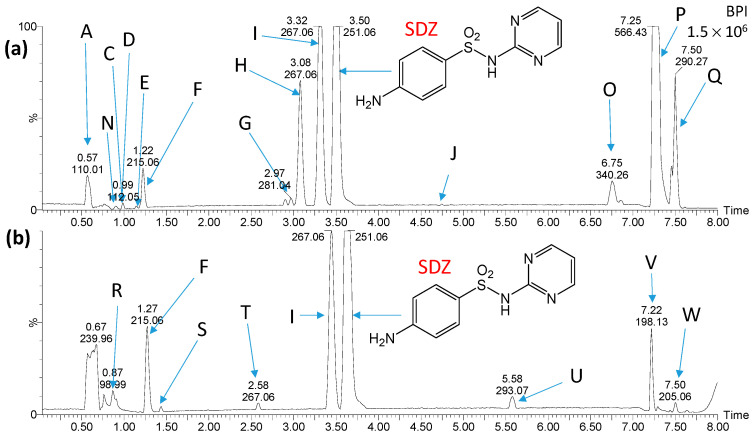
Chromatograms of the solution containing products of SDZ photocatalytic degradation after mixing with AS suspension (**a**) and after aeration of the solution for 28 days (**b**). Peaks marked with letters A, N, O, P, Q, V and W were recorded in AS samples. Structures of compounds marked with the letters C, D, E, F, G, H, I, J, R, S, T and U are shown in Figure 6.

Figure 4 and Figure 5 show chromatograms of the mixtures obtained shortly after mixing the solution containing products of SDZ photocatalytic degradation and water from the Przemsza River or AS, as well as after aeration for 28 days. Peaks marked with letters A, B, K, L, and M (Figure 4) were also identified on chromatograms obtained for the reference solution with river water (no added solution after SDZ photodegradation). On the chromatograms for the reference solution with AS, there were compounds marked with letters A, N, O, P, Q, V, and W (Figure 5); thus, they are not SDZ degradation products.

**Figure 6:**
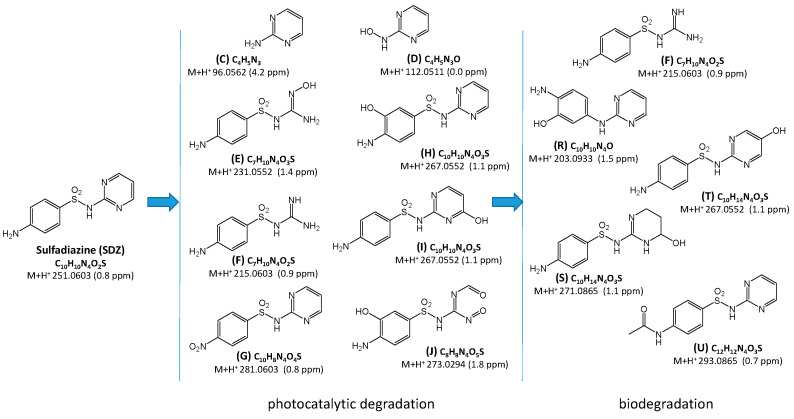
Proposed structural and molecular formulas of the products identified in the SDZ solution after photocatalytic and biological processes. Letter symbols correspond to the elution order and are consistent with the symbols in Figure 4 and Figure 5.

Proposed structural and molecular formulas of SDZ degradation products whose chromatographic peaks were identified are presented in Figure 6.

Based on the analysis of chromatograms (Figure 4 and Figure 5), eight products of SDZ photocatalytic degradation were identified (product T was likely present in trace amounts, and therefore the peak at a retention time of 2.58 min is not visible in Figure 5a). These compounds were formed by the hydrolysis of the amide group (C, D), oxidation of amine groups (D, G, E, J), ring hydroxylation (G, I, T), and by opening of the diazine ring (E, F, J). Peaks corresponding to compounds marked as E, G, H, I, and J were significantly reduced or completely disappeared during aeration for both the river water and the AS. During aeration, the amount of compounds F and T increased (Figure 5b). Moreover, only in the case of AS, peaks corresponding to compounds C and D additionally disappeared, while the formation of compounds marked as R, S, and U was recorded. These new compounds resulted from the desulfonylation (R), hydroxylation (S, T), and acylation of the amino group (U). Most likely, they were products of the biodegradation of SDZ and/or photodegradation intermediates. The presence of these products may also suggest that SDZ is slightly biodegradable in the presence of AS.

It should be noted that the products of SDZ photocatalytic degradation were much more susceptible to biodegradation compared to SDZ only, both in river water and in the presence of AS.

**Figure 7:**
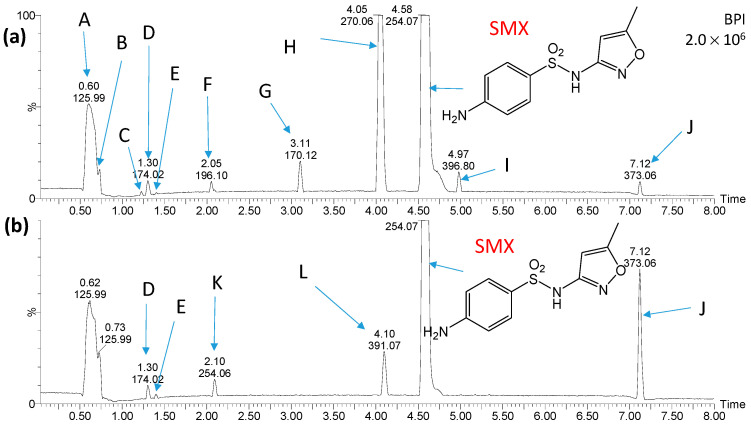
Chromatograms of the solution containing products of SMX photocatalytic degradation shortly after mixing with river water (**a**) and after aeration of the solution for 28 days (**b**). Peaks marked with letters A, B, and I, were recorded in river water samples. Structures of compounds marked with the letters C, D, E, F, G, H, J, K and L are shown in Figure 9.

**Figure 8:**
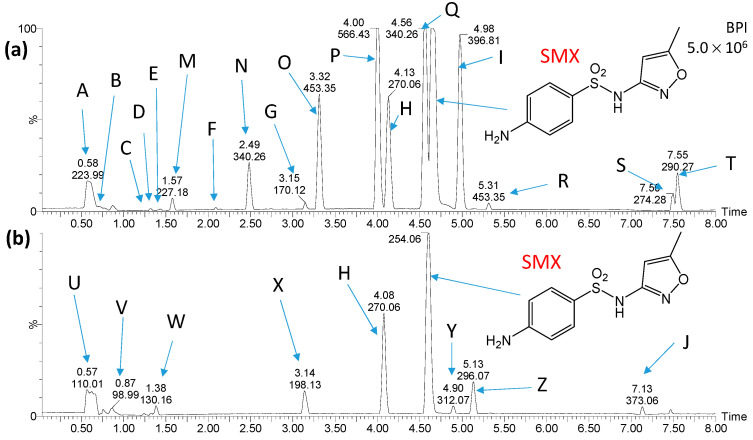
Chromatograms of the solution containing products of SMX photocatalytic degradation after mixing with AS suspension (**a**) and after aeration of the solution for 28 days (**b**). Peaks marked with letters A, B, I, M, N, O, P, Q, R, S, T, U, V, W and X were recorded in river water samples. Structures of compounds marked with the letters C, D, E, F, G, H, J, Y and Z are shown in Figure 9.

Chromatograms of mixtures of SMX photodegradation products and water from the Przemsza River or AS obtained immediately after mixing and after aeration for 28 days are presented in Figure 7 and Figure 8. Comparison of the chromatograms with those for the reference solutions revealed that the compounds marked as A, B, I, M, N, O, P, Q, R, S, T, U, V, W, and X are most likely not photocatalytic degradation products of SMX.

Seven compounds were identified as products of SMX photocatalytic degradation. These compounds formed as a result of the hydrolysis of the amide group (C, D) and hydroxylation (E, F, H, K). The structure of compound F suggests that an elimination of the amine and sulfonamide groups may have occurred, as well as hydrogenation. Moreover, the compound marked as J was the product of the attachment to the amino group of the oxidized six-membered ring. The structure of substance G was not determined.

**Figure 9:**
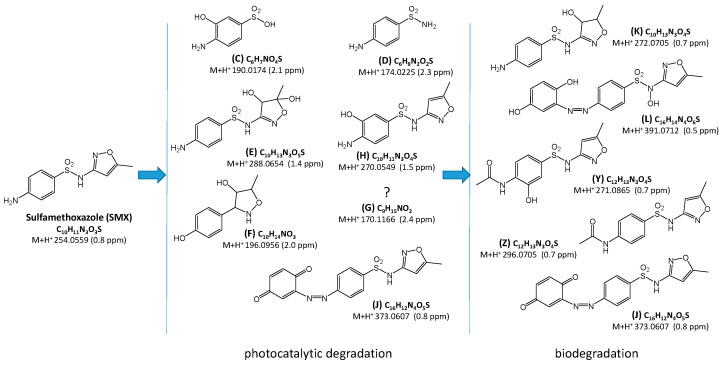
Proposed structural and molecular formulas of the products identified in the SMX solution after photocatalytic and biological processes. Letter symbols correspond to the elution order and are consistent with the symbols in Figure 7 and Figure 8.

During the aeration process of the mixtures with river water and AS suspension, a decrease in concentration or complete elimination of most of the products of SMX photocatalytic degradation, such as C, F, G, and H, was observed (Figure 7 and Figure 8). On the other hand, the amount of product J was significantly increased during the process. New compounds resulting from the transformation of the amino group, including acylation (Y and Z) or formation of azo compound (L), were also detected (Figure 9).

In a number of currently published papers on the removal of SNs from aqueous solutions, the degradation pathways of these drugs are also proposed. There are described chemical and physicochemical processes predominantly resulting in S-N bond cleavage. Then, the products of SN degradation are sulfanilamide (D, Figure 9), sulfanilic acid, and a heterocyclic amine (C, Figure 6) [26,28,40,41,42,43,44]. These compounds can also be formed by the biodegradation of SNs [45,46,47,48,49]. The products of the hydroxylation of the benzene ring (H, Figure 6 and Figure 9) and/or heterocycle ring (I, Figure 6; E, Figure 9) are also frequently identified [26,28,30,31,41,42,43,44,50,51,52,53]. Wang et al. [49] and Wang and Wang [45] described hydroxylation products of the heterocyclic ring after the biodegradation of SNs. The products of the abiotic oxidation of the amine group were also determined (G, Figure 6) [22,26,31,43,44,54]. Desulfonation of SNs (R, Figure 6) can occur by abiotic [26,43,44,50,54,55] and biological [56] processes. The heterocyclic ring-opening products of SDZ (E and F, Figure 6) were characterized by Hayati et al. [40], as well as Yang and Che [43]. Furthermore, azo compounds were identified among the products of the chemical and photocatalytic degradation of SNs [35,50]. However, we propose products containing an azo bond (J and L, Figure 9), which have probably not been described to date.

Many authors indicate the possibility of the biologically initiated acylation of nitrogen N4 of SNs [45,49,50]. For instance, products U (Figure 6) and Y, and Z (Figure 9), which we identified, had already been found. On the other hand, the degradation products of SDZ—compounds J, R, and S (Figure 6), have likely not been described to date.

The SN degradation products of subsequent chlorination and biodegradation processes were determined by Wang et al. [57] and Qin et al. [58]. However, the identification of SN transformations following photocatalysis and subsequent biodegradation is most likely a novelty.

The results of the presented studies suggest that the products of SN photocatalytic degradation can biodegrade during contact with AS and in natural surface waters. Most likely, the biodegradation resistance of the starting drugs is irrelevant. Most products formed by biodegradation do not have a free amine group at the aromatic ring, and therefore do not have antibacterial properties.

The lower intensity of the chromatographic peaks recorded after biodegradation may also indicate that the photocatalysis products underwent further degradation to simple aliphatic and mineral compounds [22,26,40,41,43,52].

### 2.4. Estimation of Ecotoxicity

**Figure 10:**
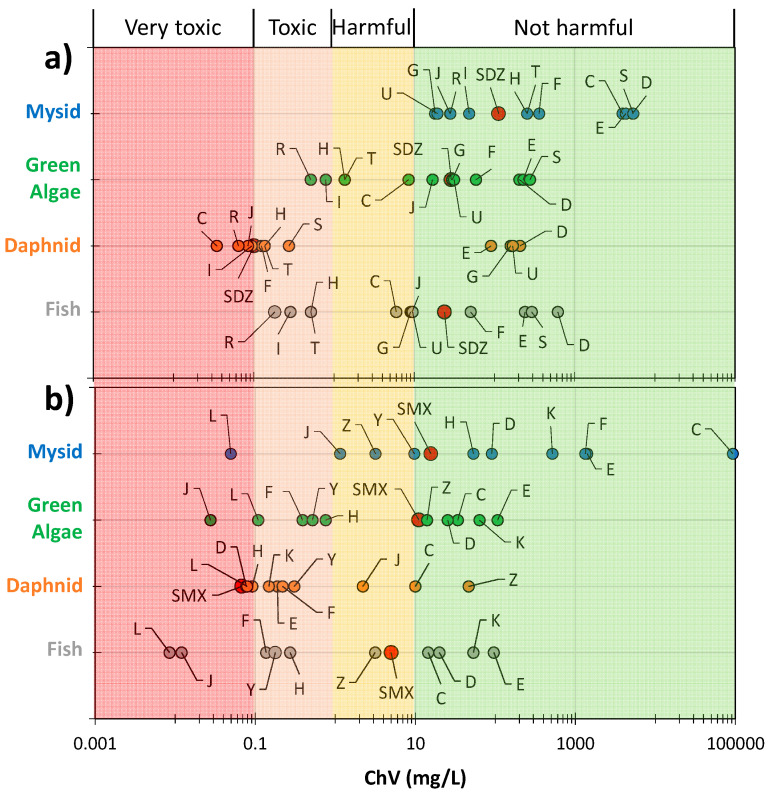
Predicted chronic toxicity of SDZ (**a**) and SMX (**b**) and their degradation intermediates. Structures of compounds marked with the letters are shown in Figure 6 and Figure 9. Colours of symbols: ●—studied antibiotics (SDZ or SMX), ●—toxicity to mysid, ●—toxicity to green algae, ●—toxicity to daphnid and ●—toxicity to fish.

The ECOSAR application was used to predict the ecotoxicity of the tested SNs and their degradation intermediates described in the previous section. Figure 10 shows the predicted chronic toxicity (ChV) to aquatic organisms from the mysid, green algae, daphnid, and fish groups. According to the criteria for the toxicity of substances to aquatic organisms established by the EU, the following categories were distinguished: very toxic, toxic, harmful, and not harmful, as shown in Table S1, Supplementary Materials.

SDZ and three products of its photocatalytic degradation (C, I, and J) as well as a biodegradation product (R) were identified as very toxic to daphnid. The toxic effect of 2-Aminopyrimidine (C) was predicted at a concentration of 0.0346 mg/L. Products F, H, S, and T were classified as toxic. Products H, I, R, and T were considered toxic to fish, while products R and I were found to be toxic to green algae. SDZ and its degradation products were not harmful to mysid (Figure 10a).

SMX was also very toxic only to daphnid (Figure 10b). Moreover, the degradation products of SMX are likely to be very toxic (J and L) or toxic (F, H, Y) to fish. The resulting photocatalytic and biodegradation azo compounds marked L and J are predicted to be toxic at concentrations of 0.00848 and 0.012 mg/L, respectively. These compounds as well as F, Y, and H were classified as toxic to green algae. Compound L was also very toxic to daphnid and mysid. Additionally, compounds D, E, F, H, K, and Y were identified as very toxic or toxic to daphnid (Figure 10b).

Xu et al. indicated the high chronic toxicity of SDZ and its photocatalytic degradation products to daphnid [59]. Furthermore, Jebalbarezi et al. [60] determined the toxicity of SMX chemical oxidation products, confirming the possibility of forming intermediates more toxic than SMX. They identified benzoquinone and aniline as the most toxic. However, these compounds were not identified in our study.

Similar conclusions on the toxicity of SN solutions after photocatalytic degradation were presented by Sapinska et al. [35].

Our results indicate that toxic intermediates can be formed during the photocatalytic oxidation of SNs. On the other hand, the results using toxicity bioassays demonstrate that the photocatalytic process reduces the toxicity of SN solutions to algae [61,62], *Daphnia magna* [63], and microorganisms [14,64]. Changes in the toxicity of the SN solutions to microorganisms after the photocatalytic process as a function of irradiation time and mineralization degree were described by Adamek et al. [14].

Our study demonstrated that highly toxic products of SNs’ transformation for aquatic organisms can also be formed by subsequent biodegradation. Changes in toxicity resulting from the biodegradation of solutions containing SNs were investigated, among others, by Kim et al. [65] and Zhang et al. [66]. Contrary to our results, they confirmed a significant reduction in the high effectiveness of removing the toxicity of the studied SN solutions using algae as indicator organisms. However, it is recommended that wastewater containing SNs, after treatment with photocatalytic methods combined with biodegradation, should be monitored for potential ecotoxicity.

## 3. Materials and Methods

### 3.1. Reagents

Two SNs were used in this study: sulfadiazine (SDZ, >99%, Sigma-Aldrich, Buchs, Switzerland) and sulfamethoxazole (SMX, >99%, Sigma-Aldrich, St. Louis, MO, USA). Commercial Aeroxide^®^ TiO_2_-P25 (Evonik, Essen, Germany) was used as a photocatalyst. In addition, a solution of sodium hydroxide at 0.1 mol/L (puriss p.a., Chempur, Piekary Śląskie, Poland), water (for LC-MS Chromasolv^®^; Fluka-Analytical, Buchs, Switzerland), acetonitrile (for LC-MS LiChrosolv^®^; Supelco, Bellefonte, PA, USA), and formic acid (98–100%, for LC-MS, LiChropur^®^; Supelco, Bellefonte, PA, USA) was used in the experiments.

### 3.2. Total Organic Carbon and Chemical Oxygen Demand Analysis

Total organic carbon (TOC) analysis was performed using the LCK380 cuvette test (HACH LANGE, Loveland, CO, USA). Chemical oxygen demand (COD) was measured using an LCK1414 cuvette test (HACH LANGE, Loveland, CO, USA). The TOC and COD results were read on a DR 3900 spectrophotometer (HACH LANGE, Loveland, CO, USA).

### 3.3. Photocatalytic Process

**Figure 11:**
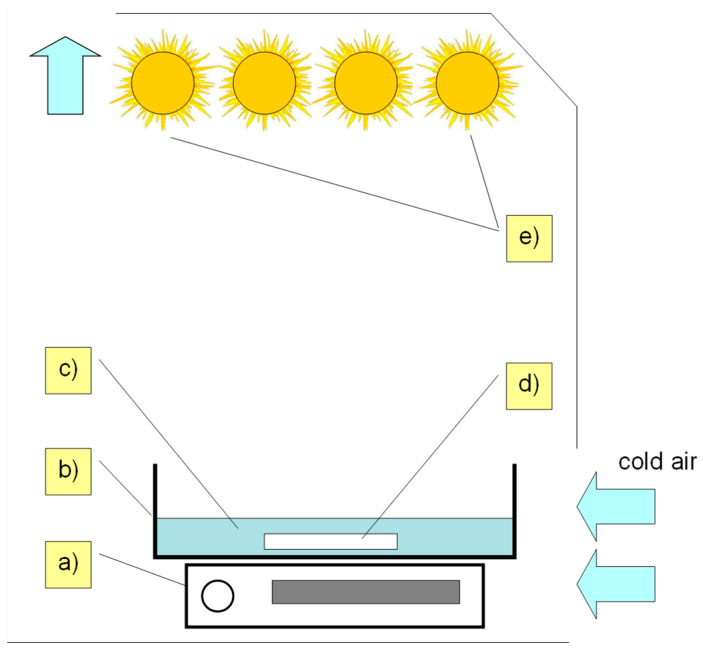
Equipment used for irradiation: (a) magnetic stirrer, (b) glass crystallizer with a capacity of 500 mL, (c) sample, (d) propeller, and (e) UVA lamps.

The solutions (0.2 mmol/L) of tested SNs were prepared in deionized water. A 100 mL solution of each SN was placed in crystallizers (500 mL), and 50 mg of solid TiO_2_-P25 was added. pH = 7.0 ± 0.5 of each sample was adjusted with a sodium hydroxide solution (1 mol/L). The obtained solutions were stirred for 10 min in the dark. Then, the mixtures were irradiated for a maximum of 120 min using fluorescent lamps (ACTYNIC BL TL 40 W/10, λ_max_ = 366 nm, Philips, Amsterdam, Netherlands) with UVa radiation at the intensity of 13.6 W/m^2^ (quantum-photo radiometer Delta OHM DO972, Caselle Di Selvazzano, Italy). The lamp spectral range is provided in the Supplementary Materials (Figure S1). The equipment used for irradiation is shown in Figure 11.

During irradiation, the temperature of the solutions was 295 ± 2 K. All mixtures were constantly stirred during irradiation. The amount of catalyst TiO_2_-P25 was determined in a preliminary test [14]. The concentrations of antibiotics used in the experiments were greater than those found in the environmental samples, but were comparable with the concentrations used by other authors [26,30,31,32,33].

At set intervals, samples were taken from the irradiated solutions, which were immediately filtered (25 mm/0.45 µm nylon syringe filter, Labfil, Zhejiang, China) and analyzed by UPLC-PDA/QTOF.

### 3.4. Biodegradation Process

The filtered solutions after the photocatalytic reaction contained about 0.06 mmol/L of unreacted SNs and their degradation products. These solutions were mixed with homogenized AS or river water in a 1:1 ratio. The AS was taken from the aerobic and denitrification chamber of the “Radocha II” WWTP (Sosnowiec, Poland). The concentration of AS (MLSS) was 3.1 ± 0.5 g/L. The river water sample was taken from the Przemsza River (collection date: 28 March 2024; conductivity = 0.579 mS/cm; pH = 7.9; turbidity = 2.9 ± 0.4 FAU; COD = 28 ± 1 mg O_2_/L; TOC concentration = 10.0 ± 0.5 mg C/L).

**Figure 12:**
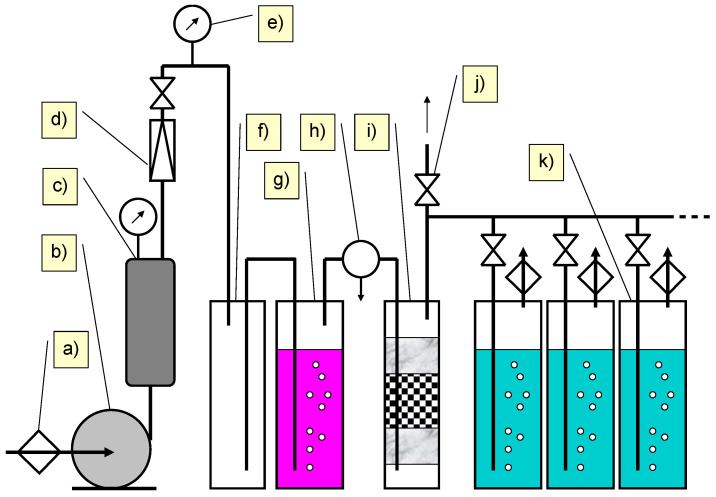
Scheme of the biodegradation test reactor: (a) HEPA filter, (b) oil-free air compressor, (c) tank, (d) pressure-reducing valve, (e) manometer, (f) safety tank, (g) gas bubbling bottles with KMnO_4_ solution, (h) droplet separator, (i) absorber with active carbon, (j) valve, and (k) bubble reactor.

The mixtures were transferred to 50 mL cylinders and then thermostated (284 ± 0.2 K) and aerated with sterile air for 28 days. The biodegradation stand is shown in Figure 12. Reference solutions without SNs were prepared and treated in an analogous manner. AS was not previously adapted in the presence of SNs.

At set intervals, samples were taken from the solutions, which were immediately filtered and analyzed by UPLC-PDA/QTOF.

### 3.5. Samples and Results Analysis

Samples were analyzed using an Acquity I Class UPLC/PDA system coupled with Xevo G2 XS QTOF (Waters, Milford, MA, USA). The degradation products of SNs were separated using an Acquity UPLC BEH C18 column, 100 × 2.1 mm (Waters, Milford, MA, USA), and the mobile gradient phase consisted of a mixture of water with 0.01% formic acid (A) and acetonitrile with 0.01% formic acid (B). Chromatographic separation parameters were as follows: flow rate 0.35 mL/min, column temperature 308 K, and sample volumes 1 and 5 µL. Contents of A in the mobile phase for SDZ: 0 min—95%, 6 min—90%, 6.5 min—50%, 7.5 min—50%, and 8.3 min—95%. Contents of B in the mobile phase for SMX: 0 min—90%, 6 min—70%, 6.5 min—50%, 7.5 min—50%, and 8.3 min—90%. The PDA detector recorded peaks at 272 nm, while the QTOF detector operated sequentially in ESI+ MS and ESI+ MS/MS modes (Table S2, Supplementary Materials).

The kinetics of SN degradation were assessed based on the peak areas recorded with a PDA detector. For each experiment, a function, C/C_0_ = f(t), and a linear regression model of the relationship, ln(C_0_/C) = f(t), were determined, where C is the SN concentration after irradiation time t and C_0_ is the initial concentration of SNs.

The calibration data for the analytical method are shown in Table 1.

The calibration curves are shown in Figures S2 and S3 in the Supplementary Materials. For each experiment, a function, C/C_0_ = f(t), and a linear regression model of the relationship, ln(C_0_/C) = f(t), were determined, where *C* is the SN concentration after irradiation time t and C_0_ is the initial concentration of SNs. Direct injections of samples and reference solution were performed. The C/C_0_ value was determined as the peak area ratio obtained for the studied solution and the reference solution (corresponding to the initial SN concentration). Sediment was not analyzed.

Molecular formulas of the degradation products of the SNs were determined based on the monoisotopic masses of molecular ions (M+H^+^) obtained using the MS/TOF technique with ESI+ ionization. The structural formulas of SN degradation products were proposed based on their molecular formulas and fragmentation spectra determined using the MS/MS/QTOF technique with ESI+ ionization, with collision energy in the range of 10–25 V. Aliphatic degradation products of SNs were not separated or identified. Compounds obtained in the reference solutions (in river water or in AS; Section 3.4) were not identified.

### 3.6. Prediction of Toxicity Using the In Silico Method

The toxicity of SNs and their degradation products in aquatic organisms were predicted using the Ecological Structure–Activity Relationship Model (ECOSAR, Version 2.2, US EPA). ECOSAR could predict, among others, the chronic toxicity of chemicals to mysid, fish, daphnid, and green algae. Details of application are available from the United States Environmental Protection Agency (US EPA) website [67].

## 4. Conclusions

The studied SNs undergo UVa-initiated photocatalytic degradation in the presence of TiO_2_-P25 according to pseudo-first-order kinetics. In solutions where irradiation was discontinued after partial degradation of the antibiotics, a total of 15 photodegradation products were identified. In the biological process, significant further degradation occurred only for SMX in the presence of AS. On the other hand, most of the photocatalytic degradation products of both investigated SNs underwent further transformations in the presence of AS, as well as in water taken from the river. During these processes, SDZ and SMX could degrade into inorganic compounds and simple aliphatic compounds. Furthermore, additional blocking of the SN pharmacophore by acyl groups may also have occurred.

From an environmental risk perspective, the combination of photocatalysis and biodegradation does not require achieving a high degree of photocatalytic mineralization of wastewater containing SNs. Photodegradation products can be efficiently removed from the environment in biological WWTPs or through naturally occurring biological processes. As a result, significant reductions in irradiation time and process costs become possible. However, our results indicate that toxic intermediates can be formed during the photocatalytic oxidation of SNs. Therefore, it is recommended that wastewater containing SNs, after treatment with photocatalytic methods combined with biodegradation, should be monitored for potential ecotoxicity.

## Figures and Tables

**Table 1:** Calibration data of UPLC methods.

Compound	Equation ^(1)^	R^2 (1)^	Linearity Range ^(1)^ (mg/L)	Limit of Quantification (LOQ) ^(1)^ (mg/L)	Limit of Detection (LOD) ^(2)^ (µg/L)
SDZ	y = 870.9x	0.9991	LOQ—125	0.107	0.075
SMX	y = 940.1x	1.0000	LOQ—127	0.102	0.075

(1) by PDA detector; (2) by QTof detector.

## Data Availability

The data presented in this study are available on request from the corresponding authors. The data are not publicity available due to the very large sizes of chromatographic files.

## Supplementary Materials

The following supporting information can be downloaded at: https://www.mdpi.com/article/10.3390/ijms25126688/s1.
