# Supplementary material for: Biodegradation of Photocatalytic Degradation Products of Sulfonamides: Kinetics and Identification of Intermediates
Source: Int J Mol Sci. 2024 Jun 18;25(12):6688. doi: 10.3390/ijms25126688 (PMC11203959; doi:10.3390/ijms25126688)
Supplement: Supplementary file 1 [file ijms-25-06688-s001.zip › ijms-3044869-supplementary.pdf]

Supplementary Materials

Biodegradation of photocatalytic degradation products of sulfonamides. Kinetics and identification of intermediates

Daria Madej-Knysak, Ewa Adamek and Wojciech Baran

Table S1. Criteria for chronic (ChV) toxicity of chemicals in the aquatic environment (based on U.S. EPA's New Chemicals Program

Classification	Chronic toxicity (mg/L)
Not harmful	ChV>10
Harmful	1< ChV <10
Toxic	0.1< ChV <1
Very toxic	ChV<0.1

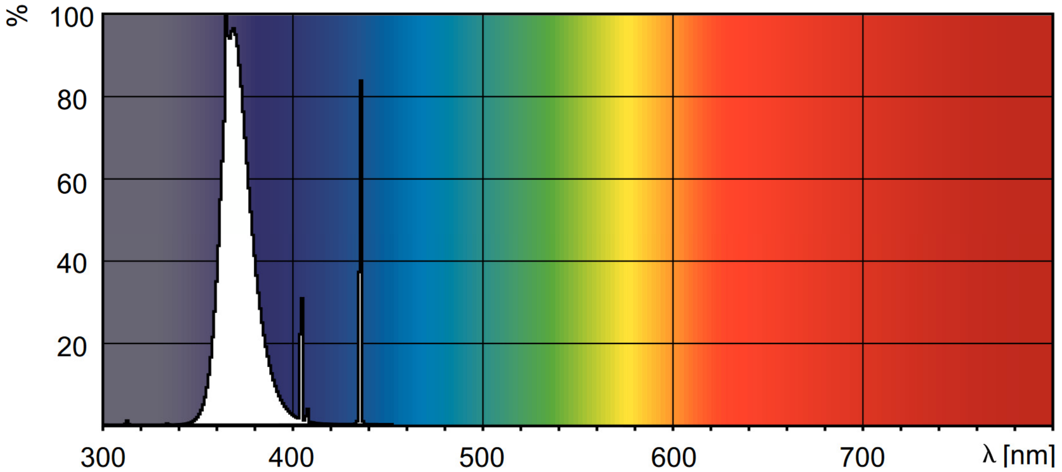

Figure S1. Spectral power distribution colour of Actinic BL TL 40W/10 lamp (available on the website: https://www.lighting.philips.com/api/assets/v1/file/PhilipsLighting/content/fp928011301020-pss-global/product_leaflet_caption.pdf)

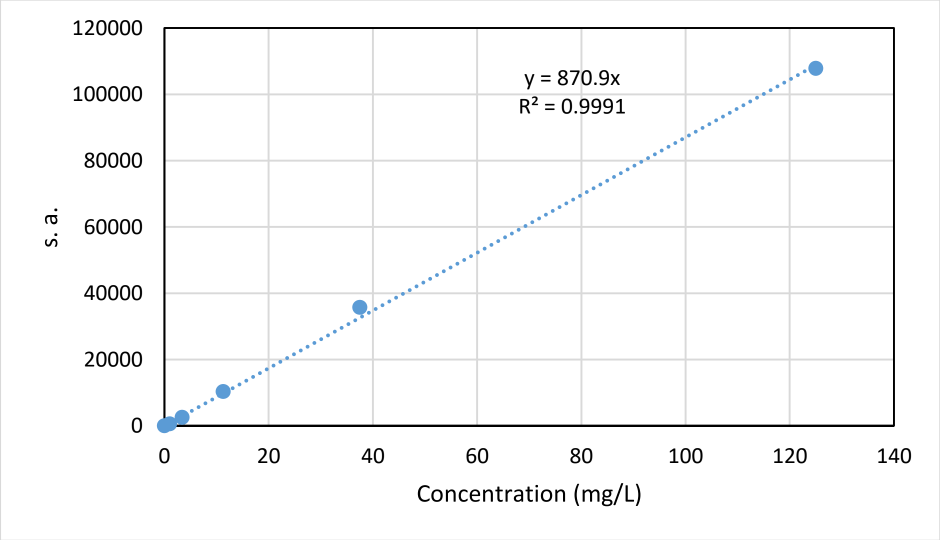

Figure S2. Calibration curve of SDZ solution (method: UPLC/PDA, λ 272 nm, sample 5 µL, t_R 3.43 min, aqueous solution)

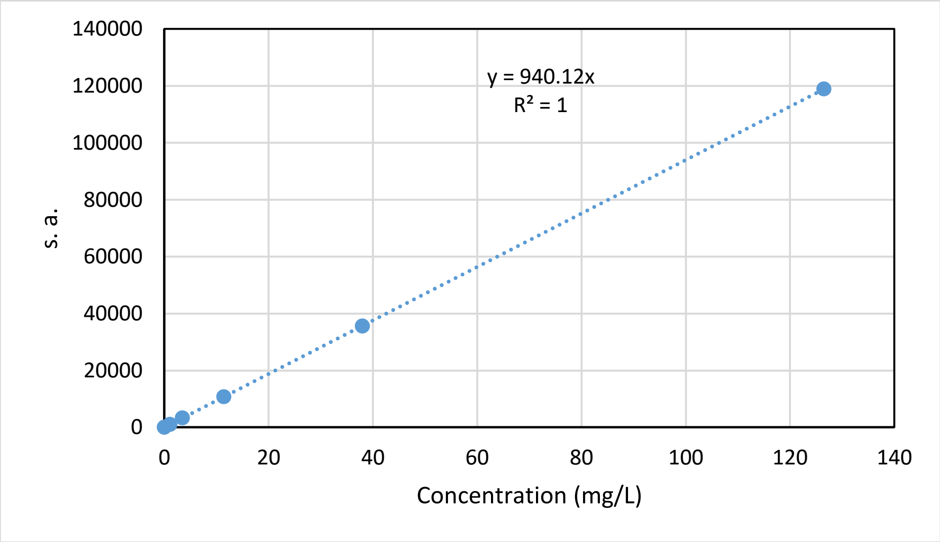

Figure S2. Calibration curve of SMX solution (method: UPLC/PDA, λ 272 nm, sample 5 µL, t_R 4.57 min, aqueous solution)

Table S2. Ionisation conditions and acquisition parameters of the Xevo Qtof detector (XEVO G2 XS) created by Masslynx v4.1

Experimental Instrument Parameters	
Polarity	ESI+
Analyser	Resolution Mode
Capillary (kV)	3.0000
Sampling Cone	40.0000
Source Temperature (°C)	100
Source Offset	80
Desolvation Temperature (°C)	250
Cone Gas Flow (L/Hr)	50.0
Desolvation Gas Flow (L/Hr)	600.0
LM Resolution	4.7
HM Resolution	15.0
Aperture 1	0.0
Pre-filter	2.0
Ion Energy	0.2
Manual Collision Energy	FALSE
Collision Energy	6.0
Detector	2825
DetectorCache	0
Sample Infusion Flow Rate (µL/min)	25
Sample Flow State	LC
Acquisition mass range	
Start mass	50.000
End mass	600.000
Scan Time (sec)	0.500
Interscan Time (sec)	0.014
Set Mass	Manual From Chromatogram
Start Mass	50.0

MSMS End Mass	600.0
Start Time (mins)	0.00
End Time (mins)	10.00
Data Format	Continuum
ADC Sample Frequency (GHz)	6.0
ADC Pusher Frequency (μs)	60.0
ADC Pusher Width (μs)	1.50
Use Tune Page Cone Voltage	YES
Use Auto Collision Energy	NO
Collision Energy (eV)	10.0-25.0
Sensitivity	Normal
Dynamic Range	Normal
Calibration	Dynamic 2
